# Genome-wide gene expression perturbation induced by loss of C2 chromosome in allotetraploid *Brassica napus* L.

**DOI:** 10.3389/fpls.2015.00763

**Published:** 2015-09-23

**Authors:** Bin Zhu, Yujiao Shao, Qi Pan, Xianhong Ge, Zaiyun Li

**Affiliations:** ^1^National Key Lab of Crop Genetic Improvement, National Center of Crop Molecular Breeding Technology, National Center of Oil Crop Improvement (Wuhan), College of Plant Science and Technology, Huazhong Agricultural UniversityWuhan, China; ^2^College of Chemistry and Life Science, Hubei University of EducationWuhan, China

**Keywords:** aneuploids, monosomy, nullisomy, transcriptome, *Brassica napus*

## Abstract

Aneuploidy with loss of entire chromosomes from normal complement disrupts the balanced genome and is tolerable only by polyploidy plants. In this study, the monosomic and nullisomic plants losing one or two copies of C2 chromosome from allotetraploid *Brassica napus* L. (2*n* = 38, AACC) were produced and compared for their phenotype and transcriptome. The monosomics gave a plant phenotype very similar to the original donor, but the nullisomics had much smaller stature and also shorter growth period. By the comparative analyses on the global transcript profiles with the euploid donor, genome-wide alterations in gene expression were revealed in two aneuploids, and their majority of differentially expressed genes (DEGs) resulted from the trans-acting effects of the zero and one copy of C2 chromosome. The higher number of up-regulated genes than down-regulated genes on other chromosomes suggested that the genome responded to the C2 loss via enhancing the expression of certain genes. Particularly, more DEGs were detected in the monosomics than nullisomics, contrasting with their phenotypes. The gene expression of the other chromosomes was differently affected, and several dysregulated domains in which up- or downregulated genes obviously clustered were identifiable. But the mean gene expression (MGE) for homoeologous chromosome A2 reduced with the C2 loss. Some genes and their expressions on C2 were correlated with the phenotype deviations in the aneuploids. These results provided new insights into the transcriptomic perturbation of the allopolyploid genome elicited by the loss of individual chromosome.

## Introduction

The aneuploidy for one species refers to the occurrence of one or more extra or missing chromosomes in the normal number (2*n*) in the cells, leading to an unbalanced chromosome complement. The loss of even one copy of one particular homologous chromosome pair (monosomy) is usually lethal for animals and diploid plants, but the gain of one (trisomy) or two (tetrasomy) copies of one chromosome is tolerable (Siegel and Amon, 2012). The exception is the sex chromosomes which show natural variation in copy number. It is long recognized that plants have better tolerance to the aneuploidy than animals. The whole set of the trisomics with one extra copy of each chromosome pair has been developed for some plants, and *Datura* trisomic plants display differing phenotypes depending on the identity of the triplicated chromosome (Blakeslee, 1922). Particularly, the complete series of monosomics and even nullisomics with the loss of one specific chromosome pair have been successfully established in some important allopolyploid crops, including the allohexaploid common wheat (*Triticum aestivum* L.), for the closely related genomes in the allopolyploid species from different progenitors can compensate functionally each other (Sears, 1954). The genetic study of the aneuploids contributes greatly to our early understanding of genome structure and homoeologous relationships between the different genomes in allopolyploids (Sears, 1954) and to the chromosome-based sequencing of the very large wheat genome (Mayer et al., 2014).

Aneuploidy should adversely affect the various aspects of normal development, because each chromosome, or chromosome pair, plays a definite role in the development of the individual, as pointed out by Boveri (1902) more than one century ago. All species including plants, animals and single-celled yeast studied respond to the aneuploidy by showing abnormalities and defects in phenotype and growth, reduced fitness and high-risk of mortality (McClintock, 1929; Singh et al., 1996; Antonarakis et al., 2004; Henry et al., 2010; Siegel and Amon, 2012). The phenomenon that the severity of growth defects scaled with the size of the trisomic chromosome was observed in all organisms studied, and an additional trend conserved across species is that monosomies (even segmental monosomies) produce more pronounced phenotype than trisomies (Siegel and Amon, 2012). Autosomal monosomies are inviable in human. Besides Down syndrome associated with Trisomy 21, the more correlations between aneuploidies, even segmental aneuploidies and many syndromes and diseases in human were elucidated continuously (Antonarakis et al., 2004; Morrow, 2010). Furthermore, as Boveri (1902) firstly proposed aneuploidy as a potential cause of cancer, structural and numerical chromosome aberrations have been revealed to be the most obvious and most distinguishing characteristics of cancer genomes (Gordon et al., 2012), and aneuploidy can predispose to tumor development (Matzke et al., 2003; Gordon et al., 2012).

It has been recognized that the aneuploidy for whole chromosome or even chromosomal segment has a severer impact on the modulation of gene expressions than the change of ploidy (Birchler and Newton, 1981; Guo et al., 1996). This might result from the disruption of strict stoichiometry of all dosage-sensitive gene products encoded by a chromosome or subset of chromosomes, proposed by the theory of gene balance (Birchler et al., 2001; Henry et al., 2006). With the high-throughput technologies (microarrays and RNA-seq) for studies of global gene expression in aneuploidy for plants and animals (Huettel et al., 2008; Zhang et al., 2010; Mäder et al., 2011; Letourneau et al., 2014), the trans-acting effects across remainder genome were found to be quite prevalent, rather than the cis-acting effects of the variant chromosomes, except for a dearth of trans-acting effects in yeast (Torres et al., 2007). Moreover, after tactfully eliminating the noise of genetic variation via a pair of monozygotic twins discordant for trisomy 21 in human, Letourneau et al. (2014) demonstrated that the differential expression between the twins was clustered in domains along all chromosomes, implying that these dysregulated domains probably involved in some symptoms of trisomy 21.

The whole-chromosome aneuploidy and structural alterations occur frequently in the nascent autopolyploids and allopolyploids (Mestiri et al., 2010; Xiong et al., 2011; Chester et al., 2012; Zhang et al., 2013), while natural species maintains a fixed and stable chromosome number. The loss and gain of chromosomes frequently involved homoeologous chromosome replacement and compensation, and the chromosome numbers at or near the euploid level were maintained possibly for dosage balance requirements. Chromosome loss and gain were also unequal across the different homologous chromosome pairs in *Brassica* and wheat (Xiong et al., 2011; Zhang et al., 2013). The important crop oilseed rape (*Brassica napus* L., 2*n* = 38, AACC) is an allotetraploid species in the *Brassicaceae* family and was formed ~7500 years ago by the natural hybridization between *B. rapa* L. (2*n* = 20, AA) and *B. oleracea* L. (2*n* = 18, CC) (Chalhoub et al., 2014). The preferential loss of some C-genome chromosomes from the complement of *B. napus* is recurrently induced in its intergeneric crosses with other crucifers (Chen et al., 2007; Du et al., 2008; Tu et al., 2010), which makes it feasible to produce aneuploids missing C-genome chromosome. One nullisomics (2*n* = 36) of *B. napus* was previously obtained from its mixploidy hybrid with the crucifer *Orychophragmus violaceus* (Hua and Li, 2006), but the identity of the chromosome lost was uncertain. The nullisomics showed the much smaller plant stature and very earlier flowering than the euploid genotype of *B. napus*. As the genomes of *B. napus* and its two extant progenitors have been sequenced (Wang et al., 2011b; Chalhoub et al., 2014; Liu et al., 2014), their genomic data available should facilitate the transcriptomic analyses of the nullisomics.

In this study, the global gene expressions of the nullisomics and the monosomics derived were investigated in comparison with original euploid *B. napus*, with the aims (1) to determine the origin of the missing chromosome, (2) to reveal the dosage effects of different copies of one particular chromosome on the transcriptomic disturbances, (3) to find the possible chromosome distribution of dysregulated domains for gene expressions, (4) to identify the molecular cause of the morphological variations associated with the chromosome loss. These findings should provide new insights into the impact of aneuploidy on genome-wide transcriptomic changes in plant allopolyploids.

## Materials and methods

### Plant materials

One nullisomics (2*n* = 36) of *Brassica napus* L. (2*n* = 38) was derived from the progenies of one mixoploidy hybrid between *B. napus* L. cv. Oro and another crucifer *Orychophragmus violaceus* (L.) O. E. Schulz (2*n* = 24) as pollen parent, by preferential elimination of the chromosomes from the latter species (Hua and Li, 2006). The identity of the lost chromosome pair remained to clarify. The nullisomics produced mainly the nullisomic progenies with the same phenotype and chromosome number (2*n* = 36) by selfing for several generations. After the euploid *B. napus* L. cv. Oro was pollinated by the nullisomics, the seed-set was good and the monosomic plants (2*n* = 37) were easily obtained for study. The plants from the selfed seeds of “Oro” and the nullisomics and putative monosomic seeds from the crossing of “Oro” and the nullisomics were established in the experimental field and greenhouse for cytological and transcriptomic analyses. The chromosome complement of the nulli-/monosomic plants was identified by cytological observations before RNA-seq.

### Cytology and FISH analyses

In order to reveal the chromosome complement of the nulli-monosomics, young ovaries from the flowering plants were collected and treated with 2 mM 8-hydroxyquinoline for 3–4 h at 22°C, and subsequently fixed in Carnoy's solution I (3:1 ethanol: glacial acetic acid, v/v) for 24 h, and stored at −20°C. For meiotic analyses, flower buds were fixed in Carnoy's solution I at room temperature, transferred to fresh mixture for 3–5 times and stored at −20°C. Cytological observation was made according to the method of Li et al. (1995).

The plasmid DNA of BAC BoB014O06 specific for C-genome of *B. oleracea* (provided by Susan J. Armstrong, University of Birmingham, Birmingham, UK) was labeled with biotin-11-dCTP by random priming using the BioPrime DNA Labeling System kit (Invitrogen, Life Technologies) according to the manufacturer's protocol (Invitrogen, Life Technologies). The probe was used to identify the C-genome chromosomes in the mono-/nullisomics. Slide preparations of chromosome for BAC-FISH were carried out mainly according to the methods of Zhong et al. (1996) and the procedures of BAC-FISH analyses followed the procedure of Cui et al. (2012). FISH analysis with this probe demonstrated that 16 and 17 chromosomes were fully labeled in the somatic cells (Figures S1A–C), and eight bivalents (8II) and eight bivalents and one univalent (8II + 1I) in pollen mother cells (PMCs) (Figures S1D–F) of the nulli-/monosomics, respectively. So the lost chromosome pair in the nullisomics belonged to the C genome.

### RNA extraction and preparation of cDNA libraries

The growth order of the leaves on the young plants in greenhouse were marked, and the newly expanded third leaves without petioles from six plants of each genotype were collected and divided into two replicates (three plants per replicate) and immediately stored in liquid nitrogen for RNA extraction. Using TRIzol reagent (Invitrogen, Life Technologies) according the manufacturer's protocol, total RNA was extracted from two biological replicates for each genotype. NanoDrop ND 1000 (NanoDrop technologies) was used to initially calculate the quality and quantity of the extracted RNA, and then the RNA Integrity Number (RIN) value was assessed by Agilent Technologies 2100 Bioanalyzer (Agilent). Only when the value of RIN was higher than 8, the RNA was used to prepare the c-DNA library according to the TruSeq RNA Sample Prep v2 protocol. Subsequently, the 100 bp paired-end reads were generated via Illumina HiSeq™ 2000.

### Mapping reads to the reference genome and gene annotations

To generate clean reads, the sequenced data were trimmed via removing adapters, low-quality sequences or bases and contaminations or overrepresented sequences. From the formulation of the sequence information of *B. napus* (Chalhoub et al., 2014), the clean reads were directly aligned to the reference genome (Brassica_napus.annotation_v5.gff3.) using Burrows–Wheeler Alignment (BWA version: 0.7.5a-r405), allowing up to 1 mismatched base. Then the genes were assembled with Cufflinks according to a reference-guided method (Trapnell et al., 2010). The differentially expressed genes (DEGs) in leaf between three different samples were computed on RNA-seq data by using CuffDiff2 (Trapnell et al., 2013). Eventually, the GO annotations for the DEGs in leaf were determined via Blast2GO (*E* < 1e-6), then the information of GO classification containing three levels was downloaded from WEGO (http://wego.genomics.org.cn/), and the graph was emerged by Orgin Pro (version 8.0).

Gene expression data are available at Gene Expression Ombinus (GEO), a database affiliated with NCBI and the accession number was GSE70400.

## Results

### Morphology of mono-/nullisomics

In comparison with the euploid *B. napus* cv. “Oro,” the nullisomics (2*n* = 36) which lost one chromosome pair from C genome presented the phenotypic change and much smaller plant architecture, including small light-green leaves, smaller plant size, shorter height, smaller flower, non-apical dominance (Figure S2). The height of its mature plants was only about half of “Oro.” Particularly, nullisomic plants flowered about 2 months earlier than “Oro,” for they produced flowers in November/December, after sown at the beginning of October in Wuhan, while the plants of “Oro” would flower in February of next year. The deviation in morphology and flowering habitat suggested that the missing chromosome pair in the nullisomics carried the genes controlling the plant height and vernalization in *B. napus*. The nullisomic plants produced short pods with several seeds after selfing, but the number of seeds in pods increased after hand-assisted pollination. Their pollen mother cells (PMCs) showed normal chromosome pairing with 18 bivalents at diakinesis and predominantly equal 18:18 segregations at anaphase I (AI) (Figure S1), and unequal 17:19 segregations at low frequency. Then the nullisomic plants produced the majority of nullisomic progeny and minority of others (2*n* = 37, 38). The nullisomic progeny which still showed the small plant size were easily distinguishable from those (2*n* = 37, 38) with much larger stature, and their rates decreased with the advance of generations. Such plants with 2*n* = 38 should be nulli-tetrasomics of *B. napus* with the same chromosome lost and another duplicated.

The monosomic plants exhibited similar morphology to normal “Oro,” but they flowered earlier about 10 days (see Figure S2). Interestingly, their euploid progeny (2*n* = 38) from selfing also tended to flower earlier, consistent with the result that some subtle developmental phenotypes of aneuploid individuals of *Arabidopsis thaliana* appeared in the diploid progeny of aneuploid parents (Henry et al., 2010). This might support the perspective that long-term phenotypic consequences of aneuploidy could persist after chromosomal balance restored (Henry et al., 2010).

### Chromosome C2 loss in the nullisomics detected by RNA-seq

To further determine the identity of the missing chromosome and to draw a comprehensive picture of the perturbations of genome-wide gene expression in the nulli-monosomics, we performed their RNA-seq and compared with the euploid. After trimmed, 38.8–74.0 million clean reads were generated from each sample. By the access to the recently released genome data of *B. napus* genotype “Darmor-bzh” (Chalhoub et al., 2014), these clean reads were directly mapped to the reference genome. Totally, 72.75–76.08% clean reads of per sample, including 27.49–30.38% multiple mapped reads, were mapped to the reference genome. Detailed information of sequencing data was summarized in Table S1.

**Figure 1:**
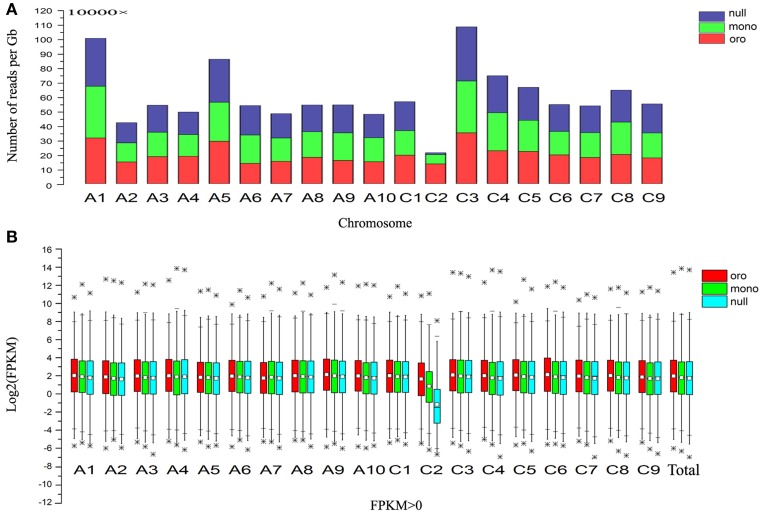
**Gene expression evidences for the loss of chromosome C2**. **(A)** Stack column of relative number of mapping reads along all chromosomes. **(B)** Box plot of Log_2_(FPKM) of the total expressed genes (FPKM > 0) and those along all chromosomes. ^*^Represents the outlier values. 1% extremely high or low value of gene expression were defined as outlier values in this study.

Then we calculated the number of reads per gigabyte along all chromosomes of three different samples to determine the origin of the missing chromosome pair (Figure 1A). Notably, compared with euploid “Oro,” the fold changes of the chromosome C2 were sharply reduced to 0.476 and 0.091 in mono/nullisomics, respectively, while the values of remainder chromosomes varied from 0.79 to 1.40 with the average 1.01 in monosomics and from 0.82 to 1.45 with average 1.06 in nullisomics. Subsequently, box plots of all expressed genes (FPKM > 0, FPKM: Fragments per Kilobase of transcript per Million mapped reads) along all chromosomes of three samples were employed to validate the expression divergence, which demonstrated that little or nothing was expressed along C2 in nullisomics (Figure 1B). From the gene expression specific for each chromosome, it was concluded that the chromosome pair C2 in *B. napus* was missing in the nullisomics.

### Global differentially expressed genes between aneuploid and euploid

**Figure 2:**
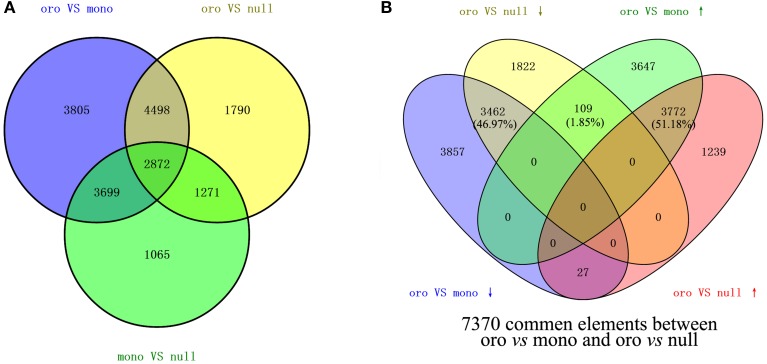
**Differentially expressed genes (DEGs) between ***B. napus*** euploid and aneuploids**. **(A,B)** DEGs among three comparisons **(A)** and 7370 common DEGs **(B)** between “Oro” vs. mono and “Oro” vs. null were shown in Venn diagram.

The values of FPKM of genes were calculated to assess the transcript expression profiling. To analyze the impact of the loss of the chromosome C2 on the global genes expressions in leaves, the CuffDiff 2 was performed to determine the DEGs (DEGs, *q* < 0.05) via evaluating the value of log_2_(fold_change) of genes. From the comparison Oro vs. monosomics, 14,874 DEGs including 7528 (50.61%) up-regulated genes and 7436 (49.39%) down-regulated genes were identified, but the up- and down-regulated ones were comparable (χ^2^ test, *P* > 0.05). In the comparison Oro vs. nullisomics, 10,431 DEGs included 5038 up-regulated genes (48.30%) and 5393 (51.70%) down-regulated (Table 1), with a significant bias to the latter (χ^2^ test, *P* < 0.05). The result that more DEGs were detected in the monomsomics than the nullisomics (14,874 vs. 10,431) when compared with the euploid was out of expectation, for the phenotype of the monosomics was similar to that of the euploid, but nullisomics was much more deviated than the monosomics from the euploid. However, only 579 DEGs (3.89%) in monosomics and 858 DEGs (8.22%) in nullisomics were directly contributed by the missing chromosome C2, which demonstrated the dominant trans-acting effects of the zero and one copy of this chromosome on the majority of DEGs in the two aneuploidies. The comparison between monosomics and nullisomics (mono vs. null) found 8907 DEGs (Table 1). From the Venn diagram of DEGs for two groups (Figure 2A), 7370 common DEGs were identified, occupying 70.65% of DEGs in Oro vs. mono and 49.55% in Oro vs. null, respectively. To gain deeper insights into transcriptional regulation of the common DEGs, we excluded those common DEGs that presented different expression patterns (up- or down-regulated) in Oro vs. mono and Oro vs. null, while, they were accounted for only 1.85% of the common DEGs. The number of co-upregulated genes was slightly higher than that of the co-downregulated ones (Figure 2B). These numerous and well conserved DEGs implied that the response to the absence of the chromosome C2 was strong in the monosomics and nullisomics.

**Table 1:** **Summary of up- and down-regulated genes in three discordant paired comparisons**.

	**Oro vs. mono**		**Oro vs. null**		**Mono vs. null**	
	**DEGs**	**Proportion (%)**	**DEGs**	**Proportion (%)**	**DEGs**	**Proportion (%)**
Up-regulated	7528	50.61	5038	48.30	3664	41.14
Down-regulated	7346	49.39	5393^*^	51.70	5243^**^	58.86
Total	14,874	–	10,431	–	8907	–

* *Chi-square test, P < 0.05*.

** *Chi-square test, P < 0.01*.

Subsequently, we excluded those DEGs along the variant chromosome C2 and calculated the shares of DEGs at different extents to reveal a detailed description that the remainder of the genome responded to zero and one copy of C2 (Table 2). These data not only showed that the number of up-regulated genes was slightly higher than down-regulated genes in both combinations, but also showed that with the increase of the fold change (FC), the up-regulated genes increasingly occupied the priority, suggesting that the remaining components of the genome probably established a mechanism against the deficiency of C2 via raising the expression of certain genes.

**Table 2:** **Summary of up- and down-regulated genes along remainder chromosomes within different fold changes**.

**FC**	**Oro vs. mono**						**Oro vs. null**					
	**Up-regulated**	**Ratio (%)**	**Down-regulated**	**Ratio (%)**	**Total**	**Ratio (%)**	**Up-regulated**	**Ratio (%)**	**Down-regulated**	**Ratio (%)**	**Total**	**Ratio (%)**
DEGs	7435	52.0	6860	48.0	14,295	–	5027	52.5	4546	47.5	9573	–
FC ≥ 1	6748	53.4	5980	47.4	12,628	88.3	4369	53.6	3788	46.4	8157	85.2
FC ≥ 5	2787	57.6	2049	42.4	4836	33.8	1596	53.9	1363	46.1	2959	30.9
FC ≥ 10	1371	56.5	1056	43.5	2427	17.0	1163	57.5	861	42.5	2024	21.1

### Distinct impacts of aneuploidy on different chromosomes

We focused on the issue that whether all retained chromosomes in the aneuploids showed random contributions of these DEGs, or certain ones and even some specific regions had preferential responses. Therefore, we calculated the proportion of genome-wide expressed genes and DEGs of the remainder chromosomes in two different paired comparisons (Table 3). The ratio [R_(DEGs/EGs)_] of DEGs to expressed genes (EGs) and ratio [R_(EGs/RGs)_] of expressed genes to referenced genes (RGs) were applied to measure the differences among chromosomes.

**Table 3:** **Proportion of expressed genes (EGs) and differentially expressed genes (DEGs) along all ***B. napus*** chromosomes**.

**Chro**	**Oro vs. mono**						**Oro vs. null**					
	**Referenced Genes (RGs)**	**Expressed Genes^d^ (EGs)**	**R_(EGs/RGs)_ (%)**	**DEGs**	**R_(DEGs/EGs)_ (%)**	**Group**	**Referenced Genes (RGs)**	**Expressed Genes^d^ (EGs)**	**R_(EGs/RGs)_ (%)**	**DEGs**	**R_(DEGs/EGs)_ (%)**	**Group**
A1	3740	1679	44.89	637	37.94	Medium	3740	1646	44.01	423	25.70	Media
A2	3717	1461	39.31	528	36.14	Medium	3717	1444	38.85	392	27.15	High
A3	6076	2728	44.90	1018	37.32	Medium	6076	2712	44.63	614	22.64	Low
A4	2973	1211	40.73	463	38.23	High	2973	1209	40.67	310	25.64	Media
A5	3750	1733	46.21	624	36.01	Low	3750	1714	45.71	436	25.44	Media
A6	4099	1948	47.52	716	36.76	Medium	4099	1923	46.91	445	23.14	Media
A7	3926	1765	44.96	706	40.00	High	3926	1751	44.60	561	32.04	High
A8	3177	1422	44.76	517	36.36	Medium	3177	1387	43.66	309	22.28	Low
A9	5728	2611	45.58	1034	39.60	High	5728	2607	45.51	662	25.39	Media
A10	3064	1440	47.00	519	36.04	Medium	3064	1446	47.19	362	25.03	Media
Ann^a^	4202	1458	34.70	551	37.79	–	4202	1465	34.86	400	27.30	–
A genome	44,452	19,456	43.77	7313	37.59	–	44,452	19,304	43.43	4914	25.46	–
C1	4498	1724	38.33	689	39.97	High	4498	1740	38.68	502	28.85	High
C2	4893	1276	26.08	579	45.38	–	4893	1194	24.40	858	71.86	–
C3	7835	3136	40.03	1119	35.68	Low	7835	3107	39.66	712	22.92	Low
C4	5696	2057	36.11	759	36.90	Medium	5696	2046	35.92	569	27.81	High
C5	5226	2184	41.79	767	35.12	Low	5226	2160	41.33	456	21.11	Low
C6	4400	1686	38.32	710	42.11	High	4400	1665	37.84	463	27.81	High
C7	5135	1934	37.66	694	35.88	Low	5135	1905	37.10	368	19.32	Low
C8	5018	1977	39.40	669	33.84	Low	5018	1944	38.74	450	23.15	Media
C9	5477	1839	33.58	683	37.14	Medium	5477	1861	33.98	443	23.80	Media
Cnn^b^	7877	2430	30.85	871	35.84	–	7877	2418	30.70	678	28.04	–
C genome	56,055	20,243	36.11	7540	37.25	–	56,055	20,040	35.75	5499	27.44	–
Unn^c^	533	72	13.51	21	29.17	–	533	74	13.88	18	24.32	–
Total	101,040	39,771	39.36	14,874	37.40	–	101,040	39,418	39.01	10,431	26.46	–

ab *Ann and Cnn represents that these genes are just speculated to belong to A genome and C genome, respectively*.

c *Unn means that these genes are not identified which sub-genome belong to (Chalhoub et al., 2014)*.

d *It should be emphasized that the expressed genes were determined only when the gene was confident in pairs, resulting in delicate difference in the number of expressed genes for the two comparisons*.

Notably, only 26.08 and 24.40% expressed genes along C2 were detected in Oro vs. mono and Oro vs. null, respectively, significantly lower than the percentages along reminder chromosomes, ranging from 33.58 to 47.52% for Oro vs. mono and from 33.98 to 47.19% for Oro vs. null (One sample *t* text, *P*_*o*/*m*_ = 4.98E-12; *P*_*o*/*n*_ = 8.35E-13). Then, we assessed the proportion of expressed genes (FPKM > 0) along all chromosomes in euploid “Oro.” Similarly, the proportion of expressed genes along C2 was the lowest (*P* = 2.03E-9), although C2 harbored a medium gene number (see Table S2). For the fact that only Trisomy 19, the smallest autosome in mouse, could evade embryonic lethal (Siegel and Amon, 2012), as well as the Trisomy 21 in humans (Antonarakis et al., 2004), it seemed possible that only nullisomic C2 in *B. napus* was survivable and stably inherited. In addition, a higher proportion (43.77% vs. 36.11% in Oro vs. mono and 43.43% vs. 35.75% in Oro vs. null) of expressed genes of A genome than that of C genome was observed in both comparisons, possibly because of the fact that much more transposable elements constituted and remolded the C genome (Zhang and Wessler, 2004; Liu et al., 2014). Nevertheless, for two pairs, no significant difference of ratio of DEGs between A and C sub-genomes was confirmed (37.59 vs. 37.25% in Oro vs. mono and 25.46 vs. 27.44% in Oro vs. null), suggesting that both A and C genome were similarly affected by the loss of C2.

Then, we assessed the value of R_(DEGs/EGs)_of remainder individual chromosomes to reveal whether certain chromosomes had preferential or suppressed contributions. Taking out the lowest C8 (33.84%) and the highest C6 (42.11%), the proportion of the DEGs of remainder chromosome showed a narrow range within 5% (35.68–40%) in Oro vs. mono. However, after excluding two extreme values, 19.32% for C7 and 32.04% for A7, the ranges raised up to over 7 (21.11–28.85%) in Oro vs. null. For the slight distinction of R_(DEGs/EGs)_value among remainder chromosomes and the discordance of contribution between two paired comparisons, it was hard to determine which chromosome invariably made a consistent contribution to the DEGs in both aneuploids. Therefore, based on the values of R_(DEGs/EGs)_ of remainder chromosomes, we divided them into three categories, low group (the first five), medium group (the intervening eight) and high group (the last five). According to this strategy, three chromosomes (C3, C5, and C7) were classified into the low group for two comparisons, suggesting these chromosomes probably were less susceptible to aneuploidy. Other three chromosomes (C1, A7, and C6) were categorized into the high group, and moreover, they invariably occupied the top three, implying that they were sensitive to aneuploidy.

### More severe perturbations of gene expressions in monosomics than nullisomics

**Figure 3:**
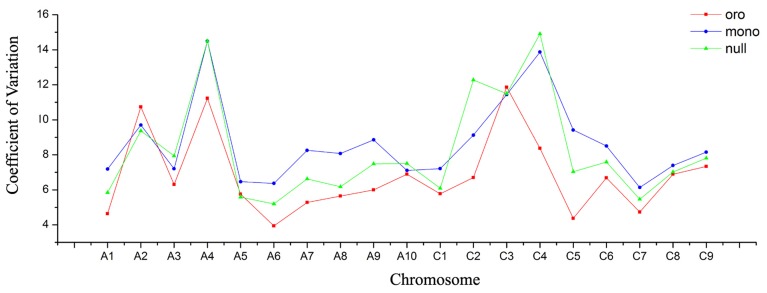
**More severe dynamic variation of gene expression in monosomics**. Coefficient of Variation (COV) of gene expression per chromosome is calculated to measure the gene expression deviation. Red panel represents for “Oro,” blue panel for monosomics, and green panel for nullisomics.

Another intriguing phenomenon was that substantially more DEGs were detected in monosomics than nullisomics. We have demonstrated that it was not the certain chromosomes which preferentially responded to the burst of DEGs in Oro vs. mono. Possibly, the whole gene expression of monosomics was more severely perturbed than in nullisomics. Then the genome-wide differential expression between the aneuploids and euploid was assessed, with a view to the distribution of the fold changes of gene expression along all chromosomes (see Figure S3). For each remainder chromosome, except some regions of certain ones, the high fold changes (|FC|> 2) of gene expressions were manifested more obviously in Oro vs. mono than Oro vs. null. Subsequently, the Coefficient of Variation (COV) of gene expression per chromosome was analyzed to compare the dynamic variation of gene expression among three types. The COV was always higher in both aneuploids, except for the chromosomes A2, C3, and A5 which showed slightly lower COV in nullisomics. Compared to nullisomics, a tendency of higher COV was observed in monosomics, except for the chromosomes A3, A4, A10, C3, C4, and the missing C2, however, the difference between them was negligible for A4 (14.493 vs. 14.496) and C3 (11.445 vs. 11.496) (Figure 3).

### Dysregulated domains along certain chromosomes

**Figure 4:**
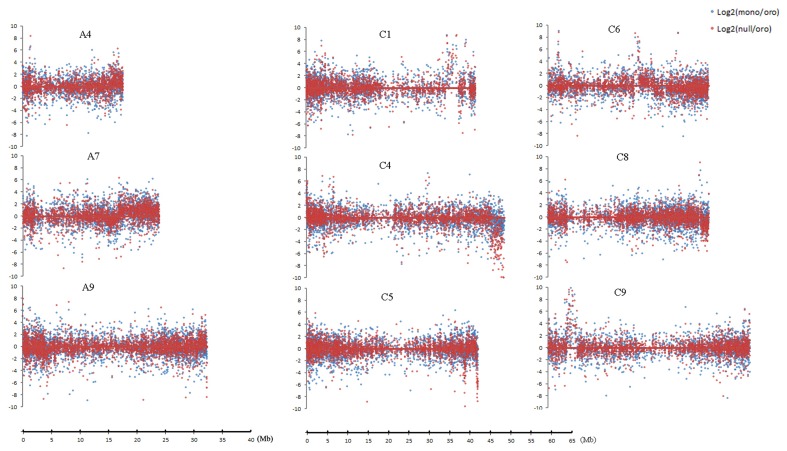
**Dysregulated domains of gene expressions on different chromosomes**. Log_2_ fold change of gene expression is performed to measure the expression deviation. Chromosomes A4, A7, A9, C1, C4, C6, and C9 harbor one domain in both comparisons, and C5 and C8 have one only in “Oro” vs. nullisomics comparison. The change between “Oro” and monosomics is shown in blue dot and change between “Oro” and nullisomics in red dot.

Several dysregulated domains of differential expressions between the aneuploids and euploid in which up- or downregulated genes obviously clustered captured our attentions, for these domains probably responded to the C2 loss. Totally, nine clear domains along distinct chromosomes (Figure 4) were revealed. These domains were either up- or downregulated in both pairs or only in one pair, but the upregulated domains consistently comprised the majority and the downregulated ones were mainly from the nullisomics. Among these domains, the minimum covered ~0.3 Mb region on C5 and the largest one located on A7 extended to ~7.2 Mb region. Remarkably, differential expressions of genes were almost up-regulated in several dysregulated domains in two aneuploids, for example, the proportion of DEGs to EGs rose to 66.67% in Oro vs. mono and 76% in Oro vs. null for the upregulated domain along C1, considerably higher than the mean expression. The detailed information of all the domains was summarized in Table 4. Interestingly, the top three chromosomes (A7, C1, and C6) occupying preferential proportion of DEGs in both paired comparisons contained one large dysregulated domains, suggesting that these dysregulated domains mainly energized the boom of DEGs.

**Table 4:** **Summary of location, size, biased regulation and proportion of dysregulated domains**.

**Chro**	**Location of domains**	**Size of domains (Mb)**	**Biased regulation**		**Proportion of DEGs of EGs**	
			**Oro vs. mono**	**Oro vs. null**	**Oro vs. mono (%)**	**Oro vs. null (%)**
A4	16.9–19.1	2.2	Up	Up	35.6	42.5
A7	16.8–24.0	7.2	Up	Up	44.5	41.7
A9	32.2–33.8	1.6	Up	Severely Up	41.0	32.6
C1	31.9–34.8	2.9	Almost up	Almost up	66.7	76.0
C4	45.6–48.9	3.3	Down	Almost down	45.0	67.5
C5	39.7–40.0	0.3	–	Down	50.0	55.6
C6	20.3–27.1	6.8	Up	Up	47.3	33.8
C8	36.4–38.4	2.0	–	Down	35.6	55.2
C9	4.6–6.8	2.2	Almost up	Almost up	71.1	74.4

### Conspicuous decline of mean gene expression for homoeologous A2

**Figure 5:**
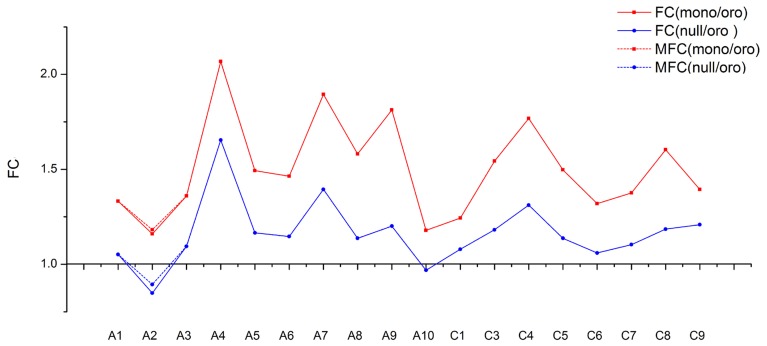
**The trends of fold change (FC) for mean genes expression along individual chromosome between “Oro” and aneuploidies**. The fold change between “Oro” and monosomics [FC(mono/Oro)] is shown in blue solid line and the fold change between “Oro” and nullisomics [FC(null/Oro)] in red solid line. Both of modified fold change (MFC) of A2 are shown in dotted line.

As the chromosomes A2 and C2 in *B. napus* were highly homoeologous chromosome pairs and were syntenic along their entire length (Parkin et al., 2005), it was fascinating how the gene expression for A2 was affected by the loss of C2, reduced or enhanced for dosage compensation? To reveal the expression differences among chromosomes, the fold changes of mean gene expression (MGE) of individual chromosomes except the absent C2 were compared between two aneuploids and euploid. The tendency of fold changes between two pairs (Figure 5) was comparatively similar (Correlation test, *R* = 0.91), consistent with above result of distributions of gene expressions, which implied a similar mechanism responding to the absence of C2 in both mon-/nullisomics again. But contrary to the expectation, the fold change of A2 turned out to be the lowest one in both pairs (1.16 in Oro vs. mono and 0.85 in Oro vs. null), and was significantly different from others of remainder chromosomes in both pairs (*P*_*o*/*m*_ = 1.17E-05, *P*_*o*/*n*_ = 1.68E-07). So the gene expression from A2 was reduced, not improved in the two aneuploids.

However, it should be pointed out that, although only uniquely mapped reads were used for analyses, some transcript profiling belonging to one chromosome were very likely assigned to its homoeolog because of sequence similarity between homoeologous pairs and the challenge for RNA-seq to completely discriminate contributions from the homoeologous transcripts. For instance, a handful of genes along C2 were detected to slightly express in nullisomics. Whether the reduction in gene expression of A2 was chiefly attributed to insufficient contributions from homoeologous C2 or to decreased expression *per se* should be taken into considerations. To eliminate the interference of insufficiency of C2, we assumed that the homoeologous genes along A2/C2 made equal contributions to each other and the transcripts profiling of C2 in nullisomics completely derived from A2 and then we introduced an adjusted value Δ to modify the MGE of A2 in deficient types. The Δ was calculated by the following formula:

$$\Delta =\frac{N_{C2}}{N_{A2}}M_{C2}$$

N_C2_ and N_A2_ represented for the number of expressed genes of C2 and A2 in “Oro,” respectively, and M_C2_ for the MGE of C2 in nullisomics. After adding Δ to MGE of A2 in nullisomics and Δ/2 to MGE of A2 in monosomics, the fold change of MGE of A2 was still significantly lower than others in both pairs (see dotted line in Figure 5, *P*_*o*/*m*_ = 1.22E-05, *P*_*o*/*n*_ = 5.75E-07). Altogether, our findings suggested that the deficiency of C2 resulted in the decreased MGE of homoeologous A2 *per se*, which implied a molecular basis for chromosomal dosage balance in *B. napus* (Xiong et al., 2011).

### Gene ontology (GO) classification of DEGs

By applying the tool of Blast2GO to determine GO annotations and to predict the functions of DEGs by classifying them into various biological processes, 14,310 (96.2%) and 9872 (94.6%) DEGs were totally annotated in Oro vs. mono and Oro vs. null, respectively. Both groups of DEGs were categorized into 52 s GO terms, except for “synapse part” and “synapse” terms, including only one same gene particularly annotated in Oro vs. null (see Table S3).

**Figure 6:**
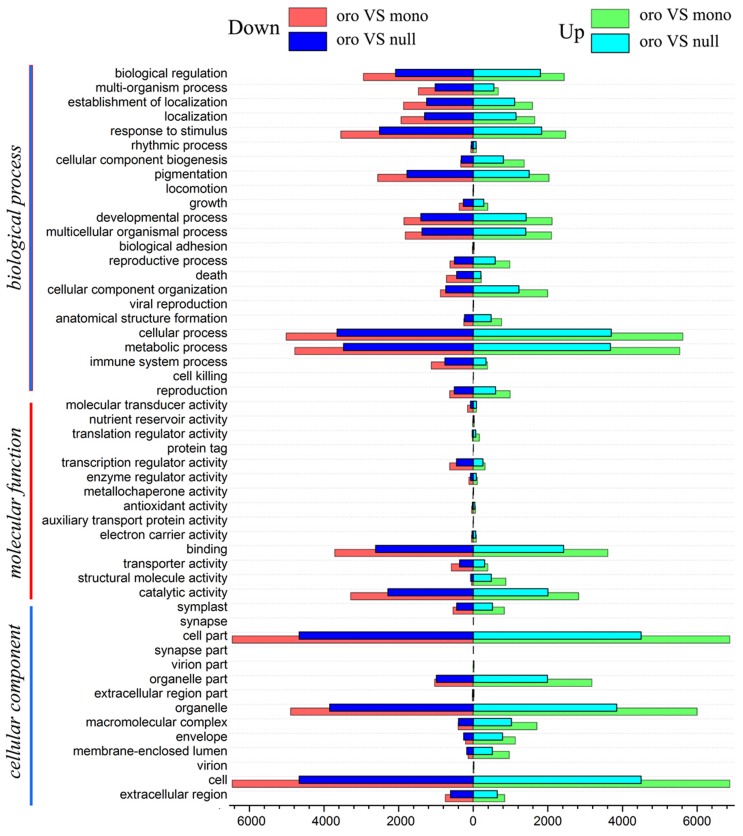
**GO assignment of annotated DEGs in “Oro vs. mono” and “Oro vs. null.”** DEGs are annotated by three categories: cellular component, molecular functions, and biological process. The left and right of *x-axis* represent the up-regulated (Red for “Oro vs. mono” and blue for “Oro vs. null”) and down-regulated genes (Green for “Oro vs. mono” and cyan for “Oro vs. null”).

Among these GO terms, “cell” and “cell part,” “catalytic activity” and “binding,” “metabolic process” and “cellular process” invariably occupied the majority for both paired comparisons, in “cellular component,” “molecular function” and “biological process,” respectively. And several terms merely contained sparse genes for both pairs, such as “protein tag” in “cellular component” and “cell killing” in “molecular function” (Figure 6). It was noteworthy that the term of “structural molecule activity” was significantly overrepresented by the up-regulated genes for both pairs (χ^2^ test, *P* < 0.01), which probably supported the view that the structure of genome was undergoing a period of instability in aneuploidy (Huettel et al., 2008; Zhu et al., 2012). Another finding for both pairs was that the downregulated genes was dominant in the term of “transcription regulator activity” (χ^2^ test, *P* < 0.01), consistent with the fact of a priority of downregulated gene expression. However, in term of “translation regulator activity,” the upregulated genes manifested booming (χ^2^ test, *P* < 0.01), strengthening the idea that some transcript expression profilings would be adjusted at the process of translation (Stingele et al., 2012). It seemed likely that the sophisticated mechanisms responding to deficiency of C2 not only functioned at the beginning of transcription but also threaded throughout the process of gene expression. Subsequently, the GO annotation of common DEGs was performed to manifest the divergence of gene expression between transcription and translation again. We noticed that despite immense gulf of DEGs number between two pairs, the proportions of DEGs of GO terms were exceedingly similar for both pairs (Correlation test, *R* = 0.9997), considering a comparatively high proportion of common DEGs and an extremely similar variation tendency of fold change of each chromosome, which further highlighted the speculation that a similar mechanism emerged to respond to the missing of C2 for both aneuploids.

### Gene expressions related to morphology in aneuploids

Considering the shorter height/non-apical dominance and earlier flowering in the aneuploids, we focused on the expression changes of those genes involved in plant hormones and signaling pathways because of its crucial role in coordination with many growth and behavioral processes in the plant life cycle, meanwhile those genes speculated to regulate the flowering time were considered as well. With the *Brassica* Database (http://brassicadb.org) and the sequencing information of *B. napus* (Chalhoub et al., 2014), 450 genes and 212 (see Table S4) genes (see Table S5) were identified for auxin and flowering time, respectively. Among the predicted genes for auxin, the gene *BnaC02g47640D* on C2 that was orthologous to *PIN4* and encoded a putative auxin efflux carrier and essential for auxin distribution in *Arabidopsis thaliana* (Weijers et al., 2005) was significantly suppressed in the nullisomics, for its expression was 4.48, 3.76, and 0 in “Oro,” mon-/ nullisomics, respectively. But no expressed genes on C2 were found for GA (GA1-GA5) synthesis and receptors (GID1a, GID1b, GID1c, and GAI) in “Oro,” mon-/ nullisomics. For those related genes on other chromosomes, the expression levels within each gene showed no significant and constant differences among three samples, except for rare one. This might suggest that the expression of these genes at early stage of plant growth was not decisive for these traits.

As the key genes responsible for flowering were *FLOWERING LOCUS C* (*FLC*), *FRIGIDA* (*FRI*), and *FLOWERING LOCUS T* (*FT*), the expression variation of these genes between aneuploids and euploid was compared, with the detection of 8 *FLC*, 4 *FRI*, and 7 *FT* paralogs in *B. napus* corresponding to their orthologs of *A. thaliana*, respectively. The *FLC* paralogs located on A2, A3, A10, C2, C3, C9 chromosomes and had two copies on A3 and C3, and their average expression was invariably maintained at comparatively low level (FPKM < 1) in three distinct types (Table 5), probably attributing to the essential condition of long daylight exposure for the accumulation of FLC proteins (Turck et al., 2008). The *FRI* paralogs distributed on A3, C3, A10, C9, but showed similar expression levels among three samples. Seven *FT* paralogs appeared on A2, A6, A7, C2, C3, C6, with two on A7. Interestingly, three *FT* paralogs (one on A7, C3, and C6) were sharply upregulated and another one on A7 markedly downregulated and the fold change of total expression of *FT* paralogs rose to 1.3 in monosomics and spectacularly to 7.2 in nullisomics (Table 5), but the other showed no or low expression. These results gave a rational explanation for the different extents of earlier flowering in mono-/nullisomics than euploid. Taken together, despite the inescapable reality of temporality and spatiality of gene expression, the expression alterations of the specific genes provided molecular mechanisms behind the respective morphological deviations associated with the loss of the chromosome C2.

**Table 5:** **Gene expression of FLOWERING LOCUS T (FT) and FLOWERING LOCUS C (FLC)**.

**Genes**	**Gene_ID**	**Gene expression (FPKM)**		
		**Oro**	**Mono**	**Null**
*FT*	BnaA07g33120D	11.04	13.46	2.63
	BnaA02g12130D	0.61	1.20	13.63
	BnaA06g21490D	0.00	0.19	0.00
	BnaA07g25310D	0.00	0.48	16.99
	BnaC02g45250D	0.47	0.00	1.59
	BnaC03g52010D	0.88	0.56	44.99
	BnaC06g27090D	0.00	1.00	14.29
	Total	12.99	16.88	94.13
	Fold change	−	1.30	7.24
	BnaA02g00370D	3.82	2.38	2.57
*FLC*	BnaA03g02820D	0.00	0.40	0.33
	BnaA03g13630D	1.50	0.41	0.27
	BnaA10g22080D	0.00	0.00	0.00
	BnaC02g00490D	0.00	0.00	0.00
	BnaC03g04170D	0.92	0.20	3.65
	BnaC03g16530D	0.19	0.00	0.11
	BnaC09g46500D	0.48	0.84	0.54
	Total	6.90	4.24	7.47
	Fold change	−	0.61	1.08

## Discussion

### Global perturbation of gene expression in aneuploids

Transcriptional changes caused by aneuploidy must be described in terms of chromosomes and/or chromosome regions with numerical changes and whether alterations in expression are in *cis* or *trans* regions, to detect whether transcriptional and protein expression changes is in direct proportion to the copy number alteration of the DNA or whether the cell minimizes the effects of aneuploidy through dosage compensation (Huettel et al., 2008; Gordon et al., 2012). In yeast and mammals, gene expression appears to correlate well with gene copy number, but the case in *Drosophila* and plants is some different. The study of gene expression in aneuploid *Arabidopsis* and maize has shown that aneuploidy alters the expression levels of several genes dispersed across the genome (Huettel et al., 2008; Makarevitch and Harris, 2010). Thus, mechanisms exist in some organisms that dampen the gene dosage imbalances caused by aneuploidy. The phenotypes associated with changes in gene copy number can not only be the result of the deregulation of the affected gene(s), but may also reflect *trans*-acting effects on other chromosomal loci or even more global alterations of the entire regulatory system (Prestel et al., 2010). In *Arabidopsis* trisomy 5, the alterations in gene expression were detected on all five chromosomes, though higher expression reflecting a dosage effect occurred on the triplicated chromosome 5 (Huettel et al., 2008). Consistently, the gene expression across every chromosome, not just chromosome 21 was revealed to be altered in human Trisomy 21, which provided molecular mechanisms behind Down's syndrome (Letourneau et al., 2014; Pope and Gilbert, 2014).

Transcriptomic change in *B. napus* aneuploidy via powerful RNA-seq technology not only determined the origin of missing chromosome, but revealed strong impact of missing copies of one chromosome on global gene expression, consistent with the other results of genome-wide perturbations of gene expression from *Arabidopsis*, fruit fly and human (Huettel et al., 2008; Malone et al., 2012; Letourneau et al., 2014). The detection of the much higher number of DEGs caused by monosomy than nullisomy (14,874 vs. 10,431) in *B. napus* was some unexpected and discordant with the phenotype performance. Subsequently, more severe perturbation of global gene expression was confirmed in monosomic individuals. These interesting results echoed with the conclusion that variable copies of individual chromosomes or chromosome segment had more detrimental effects on the phenotype than the altering of complete set chromosomes, as well as more modulation of gene expression (Birchler and Veitia, 2007).

As the A and C genomes in *B. napus* descended from a common ancestor and underwent a recent event of genome triplication special to *Brassica* genus (Paterson et al., 2004; Wang et al., 2011b; Cheng et al., 2013; Liu et al., 2014), the highly duplicated nature of the ancestral genomes should further reinforce the genomic and gene expression plasticity in the derived allotetraploid (Jackson and Chen, 2010) and led to the formation of more complex gene expression and regulatory networks in *B. napus*. According to the hypothesis of gene balance, haploinsufficiency of the whole chromosome C2 could result in a perturbation of stoichiometric relationships between gene products and disturb the regulatory networks of gene expression, giving rise to severe genomic imbalance. However, it was conceivable that a multitude of homeologous genes independently contributed to the biological functions and to the viability of the nullisomcs. Therefore, when the C2 was lost completely, some regulatory interactions and gene expression networks involved by the genes along C2 was likely to replace by the available substitutes, due to independent function on biological progress of homoeologs. Then the genomic incompleteness of the nullisomics would be recovered to some extent. So we thought the hypothesis of genomic balance elucidating the distinction between chromosome variation and ploidy change (Birchler and Veitia, 2007) appeared to be valid for the explanation of the present results.

### Compensatory upregulation of certain genes of remainder genome

**Figure 7:**
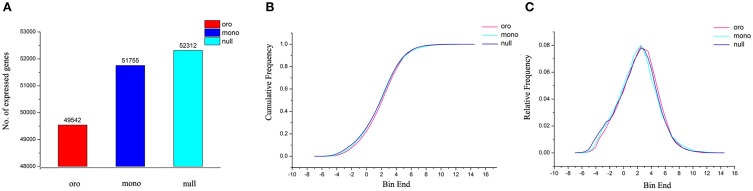
**Gene expression compensation of remainder chromosomes in aneuploides**. **(A)** The number of expressed genes (FPKM > 0) along remainder chromosomes. **(B,C)** Cumulative frequency **(B)** and relative frequency **(C)** for expressed genes [log_2_(FPKM)] are calculated by dividing gene expression into 26 expression bins.

We noted that, after taking out expressed genes (FPKM > 0) along variant C2, the number of expressed genes of remainder genome significantly increased with the reduction on C2 and the trend was much stronger in monosomics (Figure 7A). Different coverage of sequencing between “Oro” (7.0) and monosomics (10.4) potentially accounted for their gap for the fact that some rare transcripts need higher depth to be detected (Tarazona et al., 2011), however, it loosely explained the difference between monosomics and nullisomics (10.6). Alternatively, the elevated expressed genes in deficient types compensated for the insufficiency of gene expression along C2. Consistent with this notion was that the distributions of relative frequency (Figure 7B) and cumulative frequency (Figure 7C) of gene expressions (FPKM > 0) were statistically indistinguishable for the three types (Kolmogorov–Smirnov test, the *P*-value were always approximate 1). Although the average of gene expression of remainder genome was compensatory upregulation in both aneuploids (the fold change was 1.53 in monosomics and 1.16 in nullisomics), the downregulated genes were dominant (11,914 vs. 18,581 in Oro vs. mono, 19,720 vs. 18,504 in Oro vs. null; χ^2^ test, *P* < 0.01), suggesting a higher variable extents of upregulated genes. To determine the bias of up- or down-regulated genes, the genes of remainder genome were classified into the low (0 < FPKM < 10), medium (10 < FPKM < 100) or high (FPKM > 100) expression levels, in comparison with those of “Oro.” For the low expression level, the upregulated genes were significantly more than downregulated genes in both pairs (χ^2^ test, *P* < 0.01). For the medium and high levels, the skew changed to downregulated genes (χ^2^ test, *P* < 0.01), but the average gene expression within the two levels were still higher in both aneuploids. All together, these evidences suggested that a compensatory mechanism by upregulation of certain genes of remainder genome formed to respond to the C2 loss at RNA level in aneuploids.

### Dysregulated domains as a general feature of aneuploidy

Down's syndrome has been presumed for decades to be mainly caused by an overabundance of the products of chromosome 21 genes. But a recent comparison of identical human twins, only one of whom has Down's syndrome, revealed the altered gene expression across every chromosome, not just chromosome 21, suggesting that an extra copy of any chromosome could disrupt general gene regulation (Letourneau et al., 2014; Pope and Gilbert, 2014). Significantly, the increased and decreased gene-expression levels alternated consistently across large chromosomal segments, called gene expression dysregulation domains (GEDDs). Furthermore, GEDDs with increased expression corresponded to otherwise repressed genomic domains, whereas GEDDs with decreased expression corresponded to domains normally characterized by active transcription. So the difference between expressed and repressed genes was diminished in people with Down's syndrome, leading to the genome-wide flattening of gene-expression levels. With a smoothing function to define the domain borders, a total of 337 GEDDs were identified in the trisomy 21 discordant twins. The sizes of these domains were from 9 kilobases (kb) to 114 megabases (Mb), with median size of 3.2 Mb. However, no such domains were elucidated from the transcriptome analysis by microarrays for trisomy 5 in *A. thaliana*, though substantial changes in gene expression occurred, primarily on the triplicated chromosome 5 but also on the four non-triplicated chromosomes (Huettel et al., 2008). In particular, most genes on chromosome 5 showed higher expression reflecting a dosage effect, but cases of apparent dosage compensation and even down-regulation were also observed. The percentage of up-regulated genes across other chromosomes was generally higher than that of the downregulated. The possible reasons for no detection of GEDDs in *Arabidopsis* were that the less sensitiveness of microarrays than RNA-seq would probably mask the actual differences of some genes between controls and testing materials (Wang et al., 2010; Mäder et al., 2011), or that the method of smoothing function was used for the data analysis of Trisomy 21 in human.

As we lacked the knowledge of the smoothing function, but defined the obviously dysregulated domains by scanning the distribution of gene expression fold changes along the chromosomes, only nine domains of up- or down-regulation were revealed on different chromosomes, and then it was impossible to find the constant pattern of domain distribution, as in down' syndrome. The GEDDs in human were speculated to be the result of extra chromosomal materials, and the similar effect on the dysregulation of gene expression of other trisomy would uphold this hypothesis. Although these discrete and independent dysregulated domains in plant and human appeared to be quite different at organizational form, we reasoned that they were likely to be a feature of transcriptome of aneuploidy. These dysregulated domains probably attributed to the more tolerance to the adverse effects of aneuploidy in plants (Siegel and Amon, 2012). Further works are needed to undermine the transcriptional changes in terms of chromosomes and/or chromosome regions in plant aneuploids. If feasible, the cell and tissue-type difference in gene expression as done in human and animal should give more information for the change pattern in plant aneuploids.

### Chromosomal location of genes and traits by nullisomics

Traditionally, the complete set of aneuploids established for one species was used to chromosomally locate the certain traits controlled by single gene or a few genes. The chromosome mapping of one trait was conveniently deduced by its simultaneous disappearance with one chromosome in one nullisomics. But the aneuploid syndromes associated with the complex relationships of trait development made the direct location difficult. From the smaller plant stature, short height and earlier flowering shown by the *B. napus* nullisomics, the chromosome C2 lost was assumed to harbor the gene(s) responsible for the plant height and flowering habitat. The dwarfism was often caused by the mutations in genes controlling the biosynthesis or signaling pathway of the plant hormones including auxin, brassinosteroids (BRs), and gibberellins (GAs). The dwarf genes, *sd1* in rice and *Rht*-*B1b* and *Rht*-*D1b* in wheat, crucial for the Green Revolution were involved in the GA biosynthesis and signaling pathways, respectively (Peng et al., 1999; Sasaki et al., 2002). The dwarf mutants in *B. rapa* and *B. napus* were caused by the mutations in a DELLA protein which functioned as the GA signaling repressor, and the gene related was located on the chromosome A6 (Muangprom et al., 2005; Liu et al., 2010). As the formation of apical dominance was regulated by the polar auxin transport (PAT) mediated by carrier-type auxin influx and efflux proteins, auxin should affect the plant height. Two types of membrane proteins in plants were involved in cellular auxin efflux: the PIN-FORMED (PIN) family and the PGP (P-glycoproteins) sub-family of ABC transporters. In *Arabidopsis* eight PIN-related sequences have been characterized (Vieten et al., 2005), *PIN4* encoded a putative auxin efflux carrier and was essential for auxin distribution (Weijers et al., 2005). As the gene *BnaC02g47640D* on C2 chromosome was orthologous to *PIN4*, its subsequent suppression of expression likely contributed to the reduced plant height of the nullisomics, while the comparable expression level in the monosomics to the euploid which was recovered by the single copy of this chromosome was partly responsible for the nearly normal stature.

The key genes controlling the vernalization and photoperiod responses were *FLC, FRI*, and *FT* (Johanson et al., 2000; Wigge et al., 2005). The gene *FLC* has only one copy in *A. thaliana*. In the ancestral *Brassica* genome prior to triplication, the genomic regions harboring *FLC* and *FRI* on two chromosomes were brought together through block rearrangements in ancestral karyotype, which were represented three times on A2, A3, and A10 in *B. rapa* (Trick et al., 2009). The paralogous regions of *B. oleracea* were on C2, C3, and C9. Thus, in *B. oleracea, FRI* mapped quite closely to *VERNALIZATION INSENTIVE 3* (*VIN3*) (in the same block as *FRI* and required for vernalization), as well as its major target *FLC*. As the genomes of *Brassica* diploids experienced the triplication event after they diverged from *A. thaliana, FLC* was expanded from one copy in *A. thaliana* to four or five in *B. rapa* and *B. oleracea* and nine or more in *B. napus* (Tadege et al., 2001; Schranz et al., 2002; Okazaki et al., 2007; Chalhoub et al., 2014). The QTL associated with flowering time variation in *B. napus* were localized on only some of these predicted chromosomes (A2, A3, C2, C3), and also on other chromosomes (Raman et al., 2013). Some of these QTLs were clustered in genomic regions on chromosomes A2, A3, C2, and C3, and one on C2 had significant effect in delaying flowering. The largest QTL for flowering time corresponding to *BoFLC2* in *B. oleracea* was also detected in the same region of C2 chromosome (Okazaki et al., 2007). So the loss of C2 chromosome in *B. napus* should result in the earlier flowering.

Two *B. oleracea FRI* orthologs (*BoFRIa* and *BoFRIb*) were identified and mapped to chromosomes C3 and C9, respectively, while the third *FRI* appeared to have been lost from C2 during evolution in the genotypes studied (Irwin et al., 2012). *BoFRIb* was highly conserved between the three sequenced genotypes, but *BoFRIa* contained polymorphic region. Among the six *BoFRIa* alleles found in diverse genotypes of *B. oleracea*, two common ones were over-represented in vegetable types with a winter annual or biennial habit. Specially, *BnaX.FRI.d* from one *B. napus* winter variety which was confirmed to be homolog of *BoFRIa* in *B. napus* had both of the deletions identified in one of the common alleles. *BnaX.FRI.d* is also present in the European winter type and Chinese semi-winter type parental lines of one mapping population (Wang et al., 2011a). As the chromosome C2 of the cultivated *B. oleracea* germplasm analyzed carried no functional *FRI* alleles, its loss seemed not responsible for the earlier flowering in the nullisomics. But the precise parents of the natural *B. napus* which was formed ~7500 years ago were unknown, and the genetic differentiations between the present cultivated types and the ancient types certainly occurred. Homoeologous exchanges (HEs) in diverse *B. napus* genotypes were most frequent between chromosomes An1-Cn1, An2-Cn2, and An9-Cn9, and contributed to the diversification of winter, spring, and Asian types of oilseed rape (Chalhoub et al., 2014). In other side, the existence of the *FLC* on C2 with major effect suggested that it was functional in regulating the flowering or interacted with other *FLC* and *FRI* loci. Because of the temporality and spatiality of gene expression and the association between the plant height and flowering, the expression alterations of the specific genes behind the respective morphological deviations should be studied at more growth stages and conditions.

Finally, aneuploids of one species can be used for the chromosome-based genome sequencing to effectively reduce the complexity of a highly redundant genome (Mayer et al., 2014). As the assembled sequences only cover 79% of the whole genome in *B. napus* including two highly homologous genomes (Chalhoub et al., 2014), the nullisomics should assist in discriminating the homoeologous set A2/C2 with extreme similarity.

## Conflict of interest statement

The authors declare that the research was conducted in the absence of any commercial or financial relationships that could be construed as a potential conflict of interest.

## Supplementary material

Table S1 **Summary of reads mapped to reference genome of *B. napus* “Darmor-bzh**.”

Table S2 **Proportions of expressed genes along all chromosomes in “Oro**.”

Table S3 **Detail information of GO terms in both comparisons**.

Table S4 **Gene expression of those identified for auxin in *B.napus***.

Table S5 **Gene expression of those identified for flowering time in *B.napus***.

Figure S1 **BAC-FISH analysis of *B. napus* euploid, monosomics and nullisomics in mitosis and meiosis**. Red signals are from C-genome-specific probe, and chromosomes are counterstained with DAPI (Blue). **(A–C)** Mitotic cells of “Oro” (2*n* = 38) **(A)**, monosomics (2*n* = 37) **(B)**, and nullisomics **(C)** (2*n* = 36) with 18, 17, and 16 C-genome chromosomes, respectively. **(D–F)** PMCs at diakinesis from “Oro”**(D)**, monosomics **(E)**, and nullisomics **(F)**, with nine bivalents, eight bivalents and one univalent (solid arrow), eight bivalents from C-genome, respectively, Bar: 5μm.

Figure S2 **Morphology of *B. napus* monosomics and nullisomics**. **(A1–A3)** Flowering plants of “Oro,” monosomics and nullisomics (from left to right). Bar: 20cm. **(B1–B3)** Flowers of “Oro,” monosomics and nullisomics. Bar: 1cm.

Figure S3 **Distribution of differential gene expression changes between two aneuploidies and “Oro” along all chromosomes**. Log_2_ fold change of gene expression is performed to measure the expression deviation. The change between “Oro” and monosomics is shown in blue dot and change between “Oro” and nullisomics in red dot.

## Abbreviations

DEGs: differentially expressed genes
PMCs: pollen mother cells
FISH: fluorescence *in situ* hybridization
FC: fold change
COV: coefficient of variation
GEDDs: gene expression dysregulation domains
HE: homoeologous exchanges.
