# Supplementary material for: Genome-wide gene expression perturbation induced by loss of C2 chromosome in allotetraploid Brassica napus L
Source: Front Plant Sci. 2015 Sep 23;6:763. doi: 10.3389/fpls.2015.00763 (PMC4585227; doi:10.3389/fpls.2015.00763)
Supplement: Table S1 — Summary of reads mapped to reference genome of B. napus “Darmor-bzh.” [file Table1.DOCX]

| Sample | Raw data | Clean data | Clean reads | Total mapped | Uniquely mapped | Multiple mapped | GC contents | High quality base |
| --- | --- | --- | --- | --- | --- | --- | --- | --- |
| oro-1 | 5.6G | 5.2G | 42317086 | 73.75% | 70.12% | 29.88% | 46% | 98.85% |
| oro-2 | 5.2G | 4.8G | 38845622 | 73.28% | 71.34% | 28.66% | 46% | 98.79% |
| mono-1 | 7.5G | 6.9G | 56080278 | 74.25% | 70.35% | 29.65% | 47% | 98.80% |
| mono-2 | 8.3G | 7.9G | 63241288 | 74.36% | 69.62% | 30.38% | 47% | 98.82% |
| null-1 | 9.7G | 9.1G | 74019196 | 76.08% | 72.51% | 27.49% | 47% | 98.51% |
| null-2 | 6.7G | 5.8G | 46873330 | 72.75% | 70.79% | 29.21% | 46% | 98.85% |

**Table S1 Summary of reads mapped to reference genome of *B. napus* ‘Darmor-bzh’**
