# Supplementary material for: Genome-wide gene expression perturbation induced by loss of C2 chromosome in allotetraploid Brassica napus L
Source: Front Plant Sci. 2015 Sep 23;6:763. doi: 10.3389/fpls.2015.00763 (PMC4585227; doi:10.3389/fpls.2015.00763)
Supplement: Table S2 — Proportions of expressed genes along all chromosomes in “Oro.” [file Table2.DOCX]

**Table S2 Proportions of expressed genes along all chromosomes in ‘Oro’**

|  | Chrosome | Reference genes | Expressed genes | Proportion |  |
| --- | --- | --- | --- | --- | --- |
|  | A1 | 3740 | 2165 | 57.89% |  |
|  | A2 | 3717 | 1963 | 52.81% |  |
|  | A3 | 6076 | 3576 | 58.85% |  |
|  | A4 | 2973 | 1586 | 53.35% |  |
|  | A5 | 3750 | 2258 | 60.21% |  |
|  | A6 | 4099 | 2510 | 61.23% |  |
|  | A7 | 3926 | 2285 | 58.20% |  |
|  | A8 | 3177 | 1836 | 57.79% |  |
|  | A9 | 5728 | 3320 | 57.96% |  |
|  | A10 | 3064 | 1866 | 60.90% |  |
|  | C1 | 4498 | 2109 | 46.89% |  |
|  | C2 | 4893 | 1915 | 39.14%^**^ |  |
|  | C3 | 7835 | 3941 | 50.30% |  |
|  | C4 | 5696 | 2655 | 46.61% |  |
|  | C5 | 5226 | 2796 | 53.50% |  |
|  | C6 | 4400 | 2129 | 48.39% |  |
|  | C7 | 5135 | 2516 | 49.00% |  |
|  | C8 | 5018 | 2553 | 50.88% |  |
|  | C9 | 5477 | 2325 | 42.45% |  |

^**^ One sample student test, P<0.01
