# Supplementary figures and images for: Genome-wide gene expression perturbation induced by loss of C2 chromosome in allotetraploid Brassica napus L
Source: Front Plant Sci. 2015 Sep 23;6:763. doi: 10.3389/fpls.2015.00763 (PMC4585227; doi:10.3389/fpls.2015.00763)

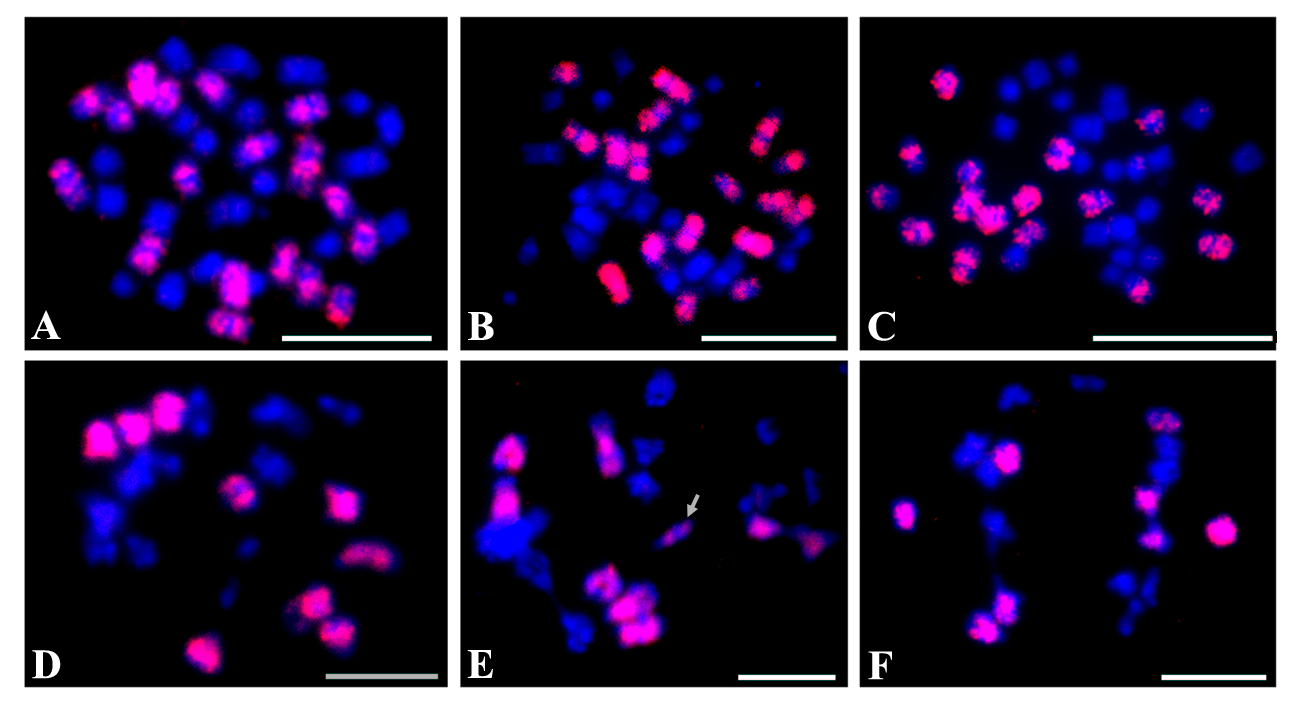

Supplement: Figure S1 — BAC-FISH analysis of B. napus euploid, monosomics and nullisomics in mitosis and meiosis. Red signals are from C-genome-specific probe, and chromosomes are counterstained with DAPI (Blue). (A–C) Mitotic cells of “Oro” (2n = 38) (A), monosomics (2n = 37) (B), and nullisomics (C) (2n = 36) with 18, 17, and 16 C-genome chromosomes, respectively. (D–F) PMCs at diakinesis from “Oro”(D), monosomics (E), and nullisomics (F), with nine bivalents, eight bivalents and one univalent (solid arrow), eight bivalents from C-genome, respectively, Bar: 5μm. [file Image1.TIF]

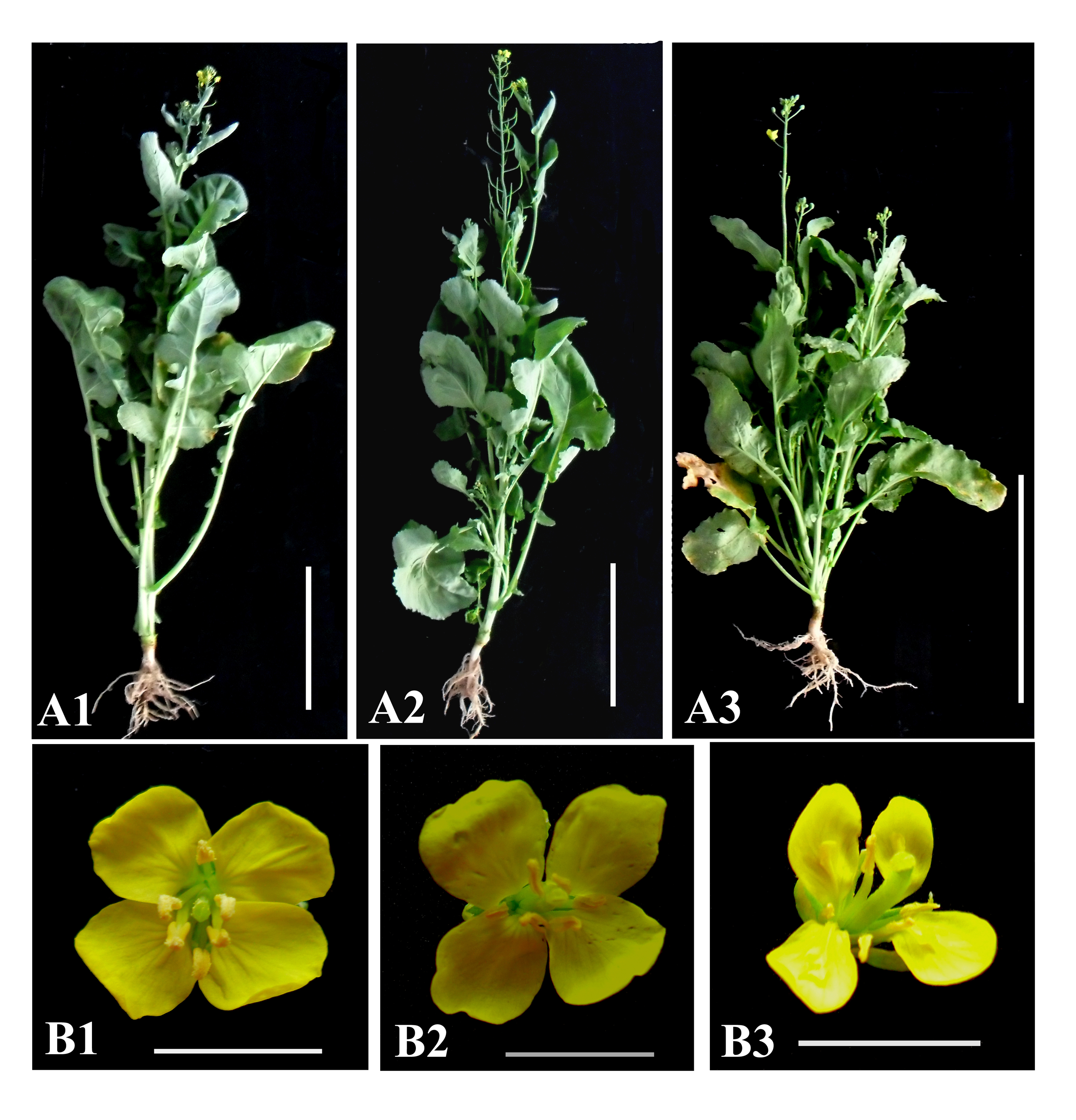

Supplement: Figure S2 — Morphology of B. napus monosomics and nullisomics. (A1–A3) Flowering plants of “Oro,” monosomics and nullisomics (from left to right). Bar: 20cm. (B1–B3) Flowers of “Oro,” monosomics and nullisomics. Bar: 1cm. [file Image2.TIF]

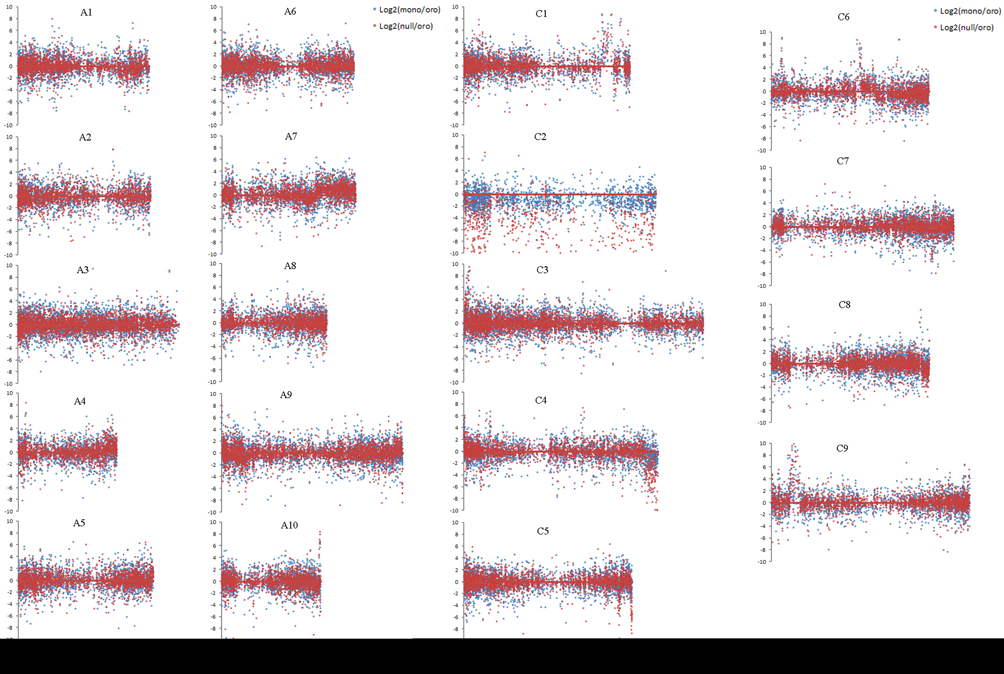

Supplement: Figure S3 — Distribution of differential gene expression changes between two aneuploidies and “Oro” along all chromosomes. Log2 fold change of gene expression is performed to measure the expression deviation. The change between “Oro” and monosomics is shown in blue dot and change between “Oro” and nullisomics in red dot. [file Image3.TIF]
